# Supplementary material for: Tissue-resident macrophages can be generated de novo in adult human skin from resident progenitor cells during substance P-mediated neurogenic inflammation ex vivo
Source: PLoS One. 2020 Jan 23;15(1):e0227817. doi: 10.1371/journal.pone.0227817 (PMC6977738; doi:10.1371/journal.pone.0227817)
Supplement: S1 Text — (DOC) [file pone.0227817.s001.doc]

**Supporting material and methods**

**Full-thickness skin organ culture**

Once the skin tissue was received, 4mm punch biopsies were obtained. Four of these punches were embedded in cryomatrix (Thermo Fisher Scientific) and immediately frozen in liquid nitrogen (day 0). The remaining punches (4 punches/condition, 2 punches/well) were cultured in a 6-well plate with 5 ml of a mixture of serum-free supplemented William’s E and RPMI medium (1:1), to better conserve the viability of immunocytes, which contains insulin, hydrocortisone and L-glutamine [1,2], and incubated at 37°C with an atmosphere of 5% CO_2_ for 48h.

After a 24h of equilibration period, skin punches were treated with 10^-8^, 10^-10^ M of SP or with a corresponding vehicle control (media only). Those doses were chosen because they are routinely used in neuroinflammation studies [1,3,4].

Alternatively, before and during SP stimulation, the selective NK1R antagonist, aprepitant [5] was administered at 10^-7^M, in order to reverse the effect of SP. To test DNA synthesis, samples were treated for 24h with 10µM EdU (5-ethynyl-2'-deoxyuridine) [6] prior to cryomatrix embedding of the tissue (Thermo Fisher Scientific). To test if the endothelial cells can be activated by SP, skin biopsies were incubated either with SP or with 2 different concentrations (0.5-50 ng/ml) of TNFα (Promega) for 24h [1].

After 24h of treatment skin punches were embedded in cryomatrix (Thermo Fisher Scientific) and snap frozen in liquid nitrogen.

**Immunohistochemistry/Immunofluorescence microscopy**

Cryomatrix-embedded tissue was processed using a cryostat to cut 7µm thick tissue section, which were collected on superfrost plus slides (Menzel Gmbh & Co KG). Tissue sections were dried for 10 min at room temperature (RT), fixed in acetone for 10 min at -20°C and rinsed in Tris-buffered saline (TBS) or phosphate buffered saline (PBS).

For detection of a single antigen by immunofluorescence (IF), tissue sections were incubated with the corresponding primary antibody (S1 Table) over night (ON) at 4°C, after appropriate pre-incubation with serum (S2 Table). Subsequently, after washing, tissue sections were incubated with the appropriate secondary antibody (S3 Table).

To perform double IF, skin sections were incubated with the appropriate dilution of the first primary antibody (S1 Table) and secondary antibodies conjugated to a fluorophore (S3 Table) for detecting the first antigen. After blocking with serum, the second primary antibody recognizing the second antigen was added (S2 Table). Following this, skin sections were then incubated with an appropriate secondary antibody (S3 Table). The primary and secondary antibodies were diluted in 10% goat serum in PBS or TBS, or in 2% goat serum in PBS or TBS, or in antibody diluent (DCS, innovative diagnostik systeme).

To detect apoptotic and proliferative cells, also commercial kits were used. For the CD68/TUNEL experiments, the detection of apoptotic cells was done using the ApopTag® Fluorescein *in Situ* Apoptosis Detection kit (Merck Millipore) following the manufacturer’s protocol.

For double-staining CD68/EdU, or triple staining CD68/CD34/EdU the EdU was detected using the EdU Click-it® Plus EdU Alexa Fluor® 594 Imaging kit (Thermo Fisher Scientific), following the manufacturer’s protocol.

Finally, if required (S2 Table), samples were washed and counterstained with 4’,6-diamidin-2’-phenylindoldihydrochlorid (DAPI, Roche) for 1 min (1µg/ml) at RT and mounted with Fluoromount-G (Southern Biotechnologies)**.**

To perform double immunohistochemistry (IHC) skin sections were pre-treated with 3% hydrogen peroxide (H_2_O_2_) (Merck Millipore) for inactivating the endogenous peroxidase (HRP) in pure methanol (JT. Baker) and/or with avidin and biotin solutions from Avidin/Biotin Vector kit (Vector Laboratories), for blocking endogenous avidin/biotin (S2 Table). Skin sections were then incubated with the appropriate dilution of the first primary antibody (S1 Table) and secondary biotinylated antibody (S3 Table), followed by the appropriate incubation with the corresponding solutions from the detection kit following the manufacturer’s protocol (S2 Table), to detect the first antigen. After, skin sections were then incubated with appropriate primary and secondary antibodies, and then with the corresponding detection kit (S2 Table). For the detection of NK1R, EnVision^+^ kit (HRP rabbit AEC) (Agilent Technologies) was used following manufacturer´s protocol (S2 Table). Finally, samples were washed, counterstained with Mayer´s Hämalaun (Carl Roth) and mounted with Faramount aqueous mounting medium (Agilent).

For fluorescence and bright field imaging, Keyence Biozero 9000 and 8100 microscopes (Keyence Corporation) were used, in combination with Nikon lenses (Nikon). The software used was Biozero-I and -II Analyzer (Keyence Corporation, Biozero-800, Higashi-Nakajima, Osaka, Japan).

**Quantitative (immuno-)histomorphometry**

Since the number of MACs is particularly prominent in the papillary dermis, close to the epidermis, where they are prone to respond at the stimuli coming from outside (7,8) this was indeed considered as reference area for the study.

Therefore, the number of single or double-positive cells were evaluated by quantitative (immuno-)histomorphometry in the papillary dermis, in an area defined as 200µm from the basement membrane of the epidermis (S1 Fig). Reference area was demarcated using Biozero-II Analyzer software.

For the evaluation of CD14^+^cells trapped in blood vessels, the number of CD14^+^MOs detected in the lumen of vessels were counted in the entire sections [9].

P-selectin expression was evaluated as immunoreactivity using National Institute of Health Image J software in the entire dermis.

For the identification of any proliferative CD68^+^ or CD34^+^, or NK1R^+^CD34^+^ cells, full skin sections were analysed.

**Supplementary References**

1. Langan EA, Vidali S, Pigat N, Funk W, Lisztes E, Bíró T, et al. Tumour Necrosis Factor Alpha, Interferon Gamma and Substance P Are Novel Modulators of Extrapituitary Prolactin Expression in Human Skin. PLoS ONE. 2013 Apr 23;8(4):e60819.

2. Lu Z, Hasse S, Bodo E, Rose C, Funk W, Paus R. Towards the development of a simplified long-term organ culture method for human scalp skin and its appendages under serum-free conditions. Exp Dermatol. 2007 Jan;16(1):37–44.

3. Ito T, Ito N, Saathoff M, Bettermann A, Takigawa M, Paus R. Interferon-gamma is a potent inducer of catagen-like changes in cultured human anagen hair follicles. Br J Dermatol. 2005 Apr;152(4):623–31.

4. Peters EMJ, Liotiri S, Bodó E, Hagen E, Bíró T, Arck PC, et al. Probing the Effects of Stress Mediators on the Human Hair Follicle. Am J Pathol. 2007 Dec;171(6):1872–86.

5. Arck PC, Handjiski B, Hagen E, Joachim R, Klapp BF, Paus R. Indications for a “brain-hair follicle axis (BHA)”: inhibition of keratinocyte proliferation and up-regulation of keratinocyte apoptosis in telogen hair follicles by stress and substance P. FASEB J Off Publ Fed Am Soc Exp Biol. 2001 Nov;15(13):2536–8.

6. Purba TS, Peake M, Farjo B, Farjo N, Bhogal RK, Jenkins G, et al. Divergent proliferation patterns of distinct human hair follicle epithelial progenitor niches in situ and their differential responsiveness to prostaglandin D2. Sci Rep. 2017 Nov 9;7(1):15197.

7. Christoph T, Müller-Röver S, Audring H, Tobin DJ, Hermes B, Cotsarelis G, et al. The human hair follicle immune system: cellular composition and immune privilege. Br J Dermatol. 2000;142(5):862–873.

8. Kashem SW, Haniffa M, Kaplan DH. Antigen-Presenting Cells in the Skin. Annu Rev Immunol. 2017 Feb 6;

9. Bagabir R, Syed F, Paus R, Bayat A. Long-term organ culture of keloid disease tissue. Exp Dermatol. 2012 Mai;21(5):376–81.
