# Supplementary figures and images for: Tissue-resident macrophages can be generated de novo in adult human skin from resident progenitor cells during substance P-mediated neurogenic inflammation ex vivo
Source: PLoS One. 2020 Jan 23;15(1):e0227817. doi: 10.1371/journal.pone.0227817 (PMC6977738; doi:10.1371/journal.pone.0227817)

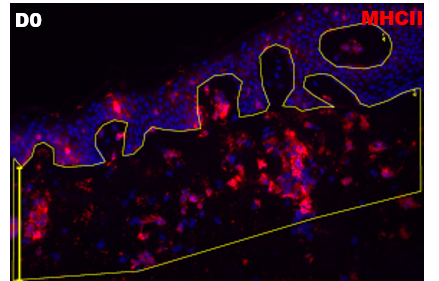

Supplement: S1 Fig — Representative pictures of the defined reference areas in the dermis (200μm from the epidermis) used for our analysis. Scale bare 100 μm. D0: day 0. (TIF) [file pone.0227817.s002.tif]

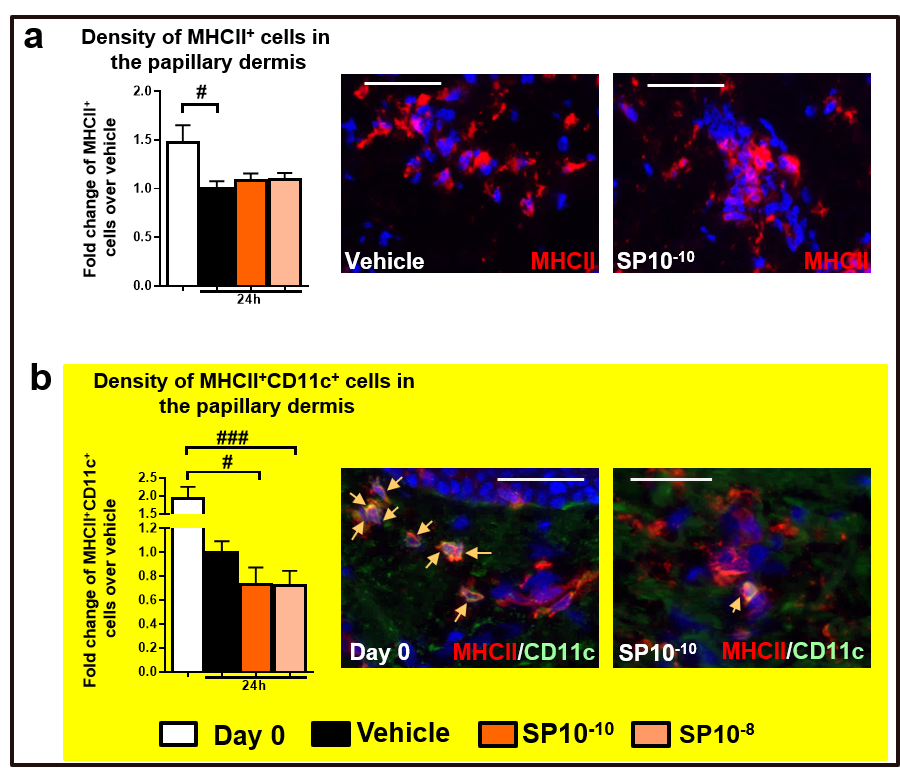

Supplement: S2 Fig — (a) Quantitative analysis, and representative images of MHCII+cells in immunofluorescence staining in human skin fragments at day 0 or treated with vehicle, or SP ex vivo. The number of cells was counted in the papillary dermis (200μm from the epidermis). N = 11–12 skin biopsies/group from 3 different donors. Fold change of Mean ± SEM, One-way ANOVA, post hoc test Bonferroni (#p<0.05). (a) Quantitative analysis, and representative images of MHCII+CD11c+cells in immunofluorescence staining in human skin fragments at day 0 or treated with vehicle, or SP ex vivo. The number of double positive cells was counted in the papillary dermis (200μm from the epidermis). N = 11–12 skin biopsies/group from 3 different donors. Fold change of Mean ± SEM, One-way ANOVA, post hoc test Bonferroni (#p<0.05; ##p<0.01). Orange arrows indicate double positive cells. Scale bare: 50μm. (TIF) [file pone.0227817.s003.tif]

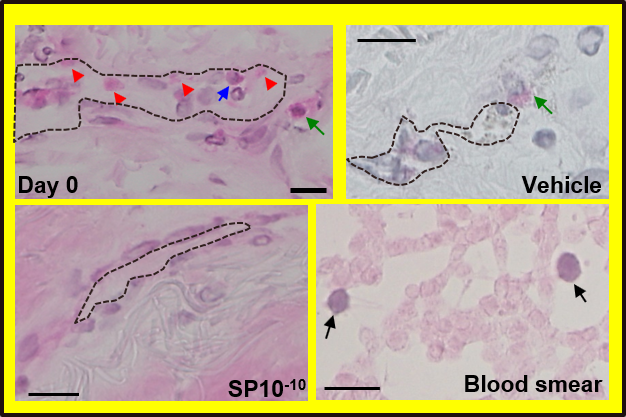

Supplement: S3 Fig — Representative pictures showing skin capillaries and blood smear control. Erythrocytes (red arrows) were visualized in few capillaries only at day 0. Intraluminal MO (blue arrow) was detected in a single lumen at day 0. Perivascular MACs (green arrows) were identify at day 0 and Vehicle control. Blood smear control showing erythrocytes and circulating T-cells (black arrows). (TIF) [file pone.0227817.s004.tif]

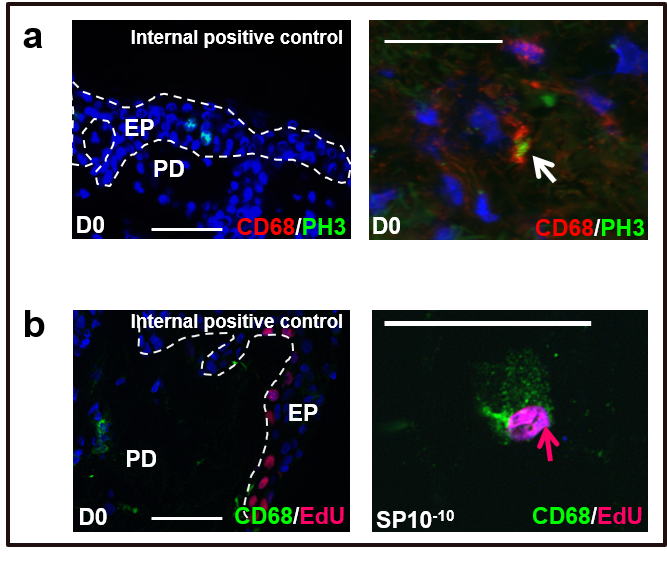

Supplement: S4 Fig — (a) Representative pictures of the internal positive control showing PH3+ cells in the epidermis. Very few PH3+CD68+MACs were visualized in D0 (day 0). This staining was qualitatively evaluated in 96 sections derived from 4 punches per conditions from 2 different donors. White arrow indicates a double positive CD68+PH3+cell (white). (b) Representative pictures of the internal positive control showing EdU+ proliferative cells in the epidermis. Very few EdU+CD68+ MACs were visualized in vehicle and SP 10-10M treated human scalp skin. EdU+CD68+ cells were detected out of 32 sections derived from 4 punches per conditions from 1 donor. Pink arrow indicates a double positive CD68+EdU+ cells. EP: epidermis; PD: papillary dermis. Scale bare 50μm. (TIF) [file pone.0227817.s005.tif]

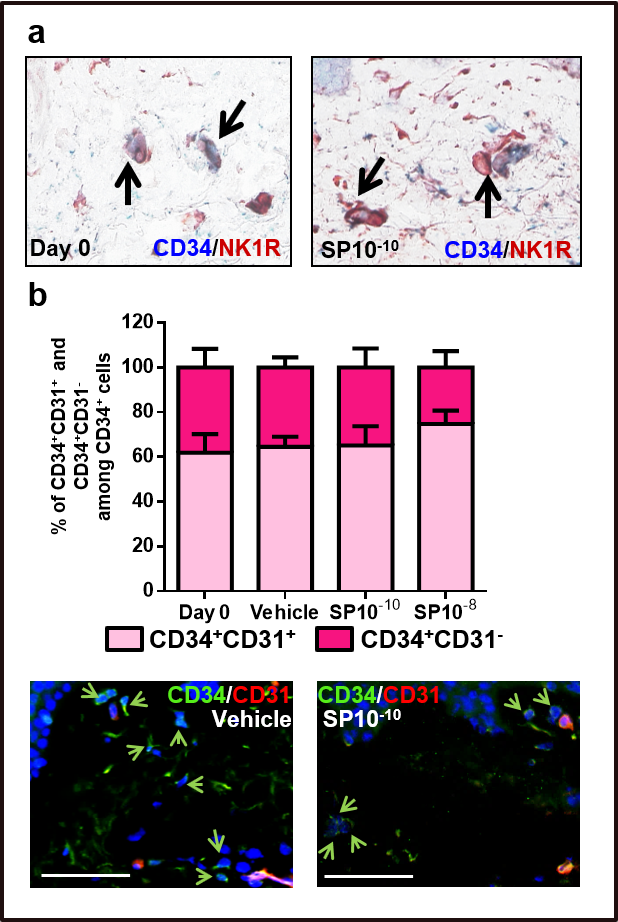

Supplement: S5 Fig — (a) Representative picture showing CD34/NK1R cells. Black Arrows indicate double positive CD34+NK1R+ cells. (b) Quantitative analysis, and representative images of CD34/CD31 in immunofluorescence staining in human skin fragments at day 0 or treated with vehicle, or SP ex vivo. The percentage of double-positive cells was counted in the papillary dermis (200μm from the epidermis). N = 7–8 skin biopsies/group from 2 different donors. Mean ± Men. Green arrows indicate CD34+CD31-cells. Scale bare: 50μm. (TIF) [file pone.0227817.s006.tif]
