# Supplementary material for: Tissue-resident macrophages can be generated de novo in adult human skin from resident progenitor cells during substance P-mediated neurogenic inflammation ex vivo
Source: PLoS One. 2020 Jan 23;15(1):e0227817. doi: 10.1371/journal.pone.0227817 (PMC6977738; doi:10.1371/journal.pone.0227817)
Supplement: S1 Table — Antibodies used for immunofluorescence stainings are listed and described in detail. (DOCX) [file pone.0227817.s007.docx]

**S1 table: Primary antibodies employed.** Antibodies used for immunofluorescence stainings are listed and described in detail.

| **Primary antibody** | **Origin** | **Clone** | **Vendor** | **Dultion** |
| --- | --- | --- | --- | --- |
| **Active Caspase 3** | Rabbit |  | Cell Signaling | 1:100 |
| **CD14** | Mouse | M5E2 | Novus Biological | 1:100 |
| **CD31** | Mouse | JC70A | Agilent | 1:30 |
| **CD34** | Mouse | 8G12 (Anti-HPCA*-2) | BD Bioscience | 1:10 |
| **CD68** | Mouse | EMB11 | Agilent | 1:50 |
| **c-kit** | Mouse |  | BD Bioscience | 1:500 |
| **Ki-67** | Mouse | Tec-3 | Agilent | 1:20 |
| **MHCII** | Mouse | CR3/43 | Agilent | 1:50 |
| **NK1R** | Rabbit |  | Abcam | 1:100 |
| **PH3** | Rabbit |  | Cell Signaling | 1:500 |
| **P-Selectin** | Sheep |  | Cell Signaling | 1:50 |
