# Supplementary material for: Tissue-resident macrophages can be generated de novo in adult human skin from resident progenitor cells during substance P-mediated neurogenic inflammation ex vivo
Source: PLoS One. 2020 Jan 23;15(1):e0227817. doi: 10.1371/journal.pone.0227817 (PMC6977738; doi:10.1371/journal.pone.0227817)
Supplement: S2 Table — Immunostaining performed and relevant details. Tris-buffered saline (TBS), phosphate buffered saline (PBS), 4’,6-diamidin-2’-phenylindoldihydrochlorid (DAPI). (DOCX) [file pone.0227817.s008.docx]

**S2 Table: List of all the immunostainings.** Immunostaining performed and relevant details. Tris-buffered saline (TBS), phosphate buffered saline (PBS), 4’,6-diamidin-2’-phenylindoldihydrochlorid (DAPI).

| **Antigen(s)** | **Preincubation/**  **Blocking** | **1^st^ detection system** | **2^nd^ detection system** | **3^rd^ detection system** | **counter staining** |
| --- | --- | --- | --- | --- | --- |
| **CD14/CD31** | 10% goat serum in TBS | Alexa Fluor^®^ 546 | Alexa Fluor^®^ 488 |  | DAPI |
| **CD14/CD68** | 10% goat serum in TBS | Alexa Fluor^®^ 546 | Alexa Fluor^®^ 488 |  | DAPI |
| **CD34** | 10% goat serum in PBS | Alexa Fluor^®^ 488 |  |  | DAPI |
| **CD34/CD31** | 10% goat serum in PBS | Alexa Fluor^®^ 488 | Rhodamine |  | DAPI |
| **CD34/Ki-67** | Avidin/Biotin Blocking Kit Vectore  3% H_2_O_2_ in Methanol | Biotin Complex – Alkaline. Phosphatase  +  Vector Blue Kit (AP) | VECTASTAIN^®^ Elite^®^ ABC-HRP Kit  +  3,3-Diaminobenzidin (DAB) |  | Mayer´s Hämalaun |
| **CD34/NK1R** | Avidin/Biotin Blocking Kit Vectore  Peroxidase Block  Envision kit | Biotin Complex – Alkaline. Phosphatase  +  Vector Blue Kit (AP) | 3-Amino-9-Ethylcarbazole (AEC) |  | Mayer´s Hämalaun |
| **CD68** |  | Alexa Fluor^®^ 488 |  |  | DAPI |
| **CD68/Active-caspase3** | 10% goat serum in TBS | Alexa Fluor^®^ 546 | Alexa Fluor^®^ 488 |  | DAPI |
| **CD68/CD34** | 10% goat serum in PBS | Alexa Fluor^®^ 546 | Alexa Fluor^®^ 488 |  | DAPI |
| **CD68/c-kit** | Pre-incubation with 1% BSA in TBS | Alexa Fluor^®^ 546 | Alexa Fluor^®^ 488 |  | DAPI |
| **CD68/EdU** |  | Alexa Fluor^®^ 488 | Alexa Fluor^®^ 594 |  | DAPI |
| **CD68/CD34/EdU** | 10% goat serum in PBS | Alexa Fluor^®^ 488 | Alexa Fluor^®^ 546 | DieBlue 350 |  |
| **CD68/ki-67** |  | Alexa Fluor^®^ 488 | Rhodamine |  | DAPI |
| **CD68/TUNEL** |  | Alexa Fluor^®^ 546 | Fluorescein |  | DAPI |
| **MHCII** | 10% goat serum in TBS | Rhodamine |  |  | DAPI |
| **MHCII/ki-67** | Pre-incubation with 1% BSA in TBS | Alexa Fluor^®^ 488 | Alexa Fluor^®^ 546 |  | DAPI |
| **PH3/CD68** | 10% goat serum in TBS +0.1% Triton | Alexa Fluor^®^ 488 | Alexa Fluor^®^ 546 |  | DAPI |
| **P-Selectin** | 10% goat serum in PBS | Rhodamine |  |  | DAPI |
