# Supplementary material for: Tissue-resident macrophages can be generated de novo in adult human skin from resident progenitor cells during substance P-mediated neurogenic inflammation ex vivo
Source: PLoS One. 2020 Jan 23;15(1):e0227817. doi: 10.1371/journal.pone.0227817 (PMC6977738; doi:10.1371/journal.pone.0227817)
Supplement: S3 Table — Fluorescein isothiocynate (FITC). (DOCX) [file pone.0227817.s009.docx]

**S3 Table: List of the secondary antibodies.** Fluorescein isothiocynate (FITC)

| **Secondary Antibody** | **Vendor** | **Dilution** |
| --- | --- | --- |
| **Goat anti-mouse Alexa Fluor^®^ 488** | Thermo Fisher Scientific | 1:400 |
| **Goat anti-mouse Alexa Fluor^®^ 546** | Thermo Fisher Scientific | 1:400 |
| **Goat anti-mouse biotinylated** | Beckman coulter | 1:200 |
| **Goat anti-mouse Rhodamine** | Jackson Immunoresearch Laboratories | 1:200 |
| **Goat anti-rabbit Alexa Fluor^®^ 488** | Thermo Fisher Scientific | 1:400 |
| **Goat anti-sheep Rhodamine** | Jackson Immunoresearch Laboratories | 1:200 |
| **Labelled Polymer-HRP anti- rabbit**  **Envision Kit** | Agilent |  |
